# Supplementary material for: Diverting glial glycolytic flux towards neurons is a memory-relevant role of Drosophila CRH-like signalling
Source: Nat Commun. 2024 Dec 2;15:10467. doi: 10.1038/s41467-024-54778-x (PMC11612226; doi:10.1038/s41467-024-54778-x)
Supplement: Supplementary file 1 — Supplementary Information [file 41467_2024_54778_MOESM1_ESM.pdf]

Supplementary Information

Supplementary Figures 1 to 8

Supplementary Tables 1 to 4

Supplementary Notes 1 and 2

Supplementary References

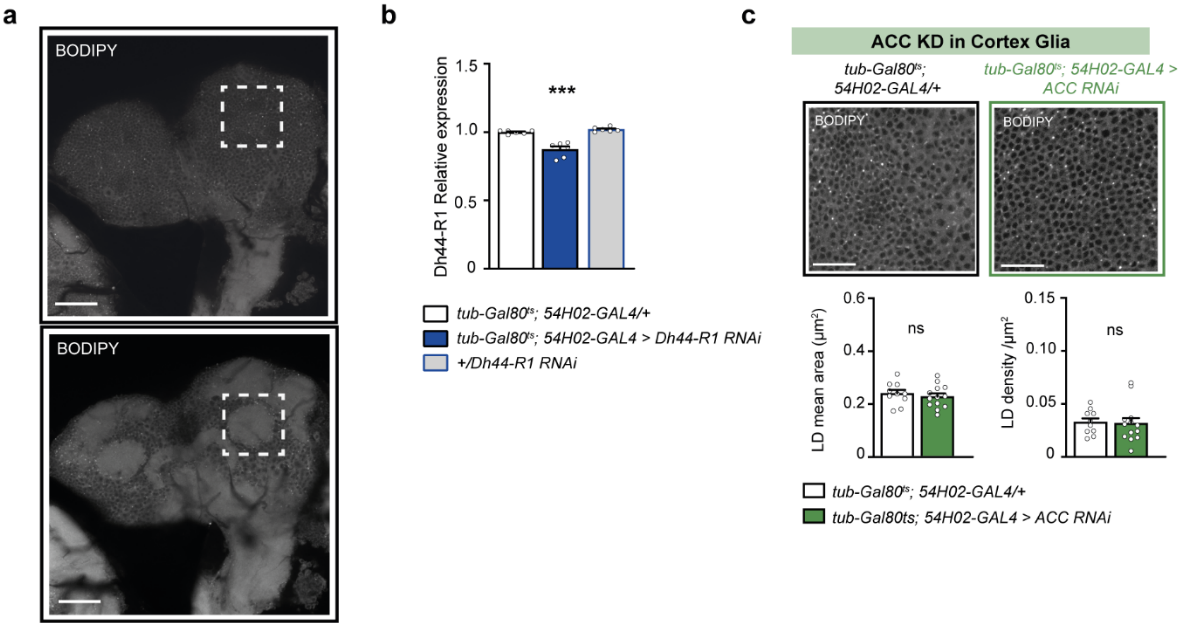

Supplementary Figure 1: Control experiments regarding the effect of Dh44-R1 on glial LD content.

(**a**) Confocal slices showing a full-size wild-type fly central brain stained with BODIPY. MB calyx (bottom panel) was used as a landmark to delimit a square ROI, that was copied on a confocal slice 4µm above. Scale bar: 50 µm. (**b**) Measurement of whole-head *Dh44-R1* mRNA by qPCR revealed reduced expression of *Dh44-R1* when the *Dh44-R1* RNAi was expressed in cortex glia (n = 6, p = 4.10^{-6}). (**c**) BODIPY staining in MB-surrounding cortex glia and quantification comparing flies expressing *ACC* RNAi in adult cortex glia to genotypic controls (n = 10;13 from left to right; mean area: $t_{21} = 0.66$, p = 0.51; density: $t_{21} = 0.185$, p = 0.85). Scale bar: 20 µm. RNAi lines KK108591 (*Dh44-R1*) and GD3482 (*ACC*) were used in this figure. Data are represented as mean ± SEM. ns: not significant, $p > 0.05$, ***$p < 0.001$ by two-tailed Student's t test (panel c) or Tukey's pairwise comparison following one-way ANOVA (panel b). Source data are provided as a Source Data file.

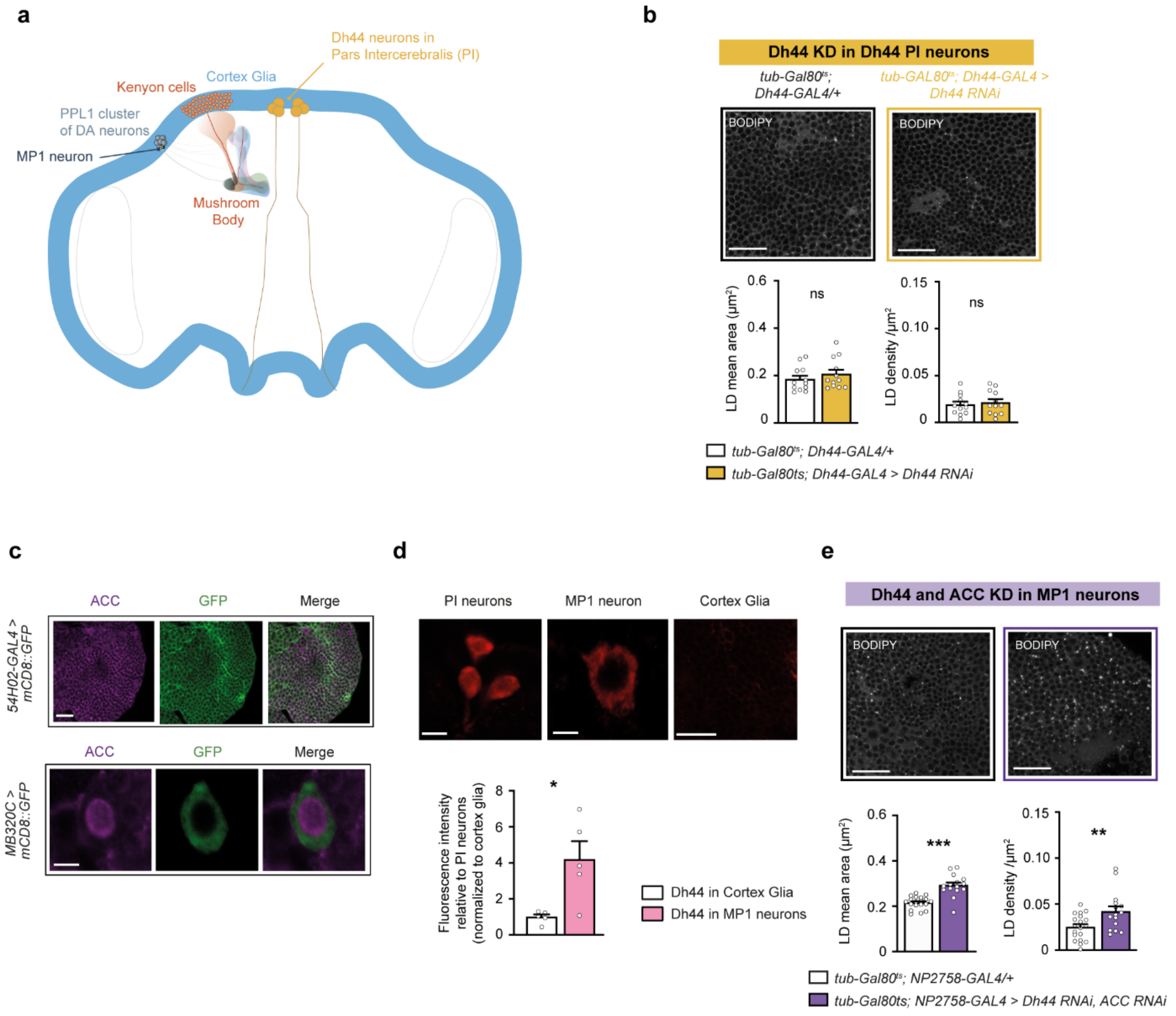

Supplementary Figure 2: Complementary experiments regarding the characterization of an MP1 neuron to cortex glia Dh44 signalling axis.

(**a**) Schematic representation of the fly brain, emphasizing cortex glia, Dh44-positive neurons in the pars intercerebralis (PI), Dh44-positive MP1 neurons within the PPL1 cluster of dopamine (DA) neurons, and. MB, the learning and memory centre.

(**b**) BODIPY staining in the MB cortex region and quantification of LD mean area and density comparing flies expressing *Dh44* RNAi in Dh44 neurons from the PI to genotypic controls.(n = 12; mean area: t_{22} = 0.90, p = 0.37; density; t_{17} = 0.54, p = 0.59). Scale bar: 20 µm.

(**c**) Immunoreactivity of the ACC antibody (magenta) in flies expressing GFP in cortex glia (top row; scale bar: 20 µm) or in MP1 neurons (bottom row; scale bar: 5 µm). There was a broad cytosolic staining of the ACC antibody throughout cortex glia. In MP1 neurons, by contrast, the cytosol did not show any

signal. Signal was present in the nucleus, which does not correspond to a reported localization of ACC (in mammals, ACC localization was reported in the cytosol and the outer mitochondria membrane[1,2], as well as the endoplasmic reticulum membrane[3]). Off-target nuclear labeling of this antibody in Drosophila cells was reported in reference [4].

(**d**) Comparison of Dh44 immunostaining in MP1 neurons and cortex glia. Dh44 neurons of the PI were used as a reference intensity value (n = 5, $t_8 = 3.122$, p = 0.01). Scale bars: PI neurons: 10 µm. Cortex glia : 20 µm; MP1 neuron : 5 µm.

(**e**) BODIPY staining in the MB cortex region and quantification of LD mean area and density comparing flies expressing both *Dh44* RNAi and *ACC* RNAi in MP1 neurons to genotypic controls. (n = 19;15; mean area: $t_{30} = 5.49$, p = 2.10^{-6}; density: $t_{32} = 2.78$, p = 0.009). Scale bar: 20 µm.

RNAi line JF01822 (*Dh44*) was used in this figure. Data are represented as mean ± SEM. ns: not significant, p > 0.05, *p < 0.05, **p < 0.01, **p < 0.001 by two-tailed Student's t test. Source data are provided as a Source Data file.

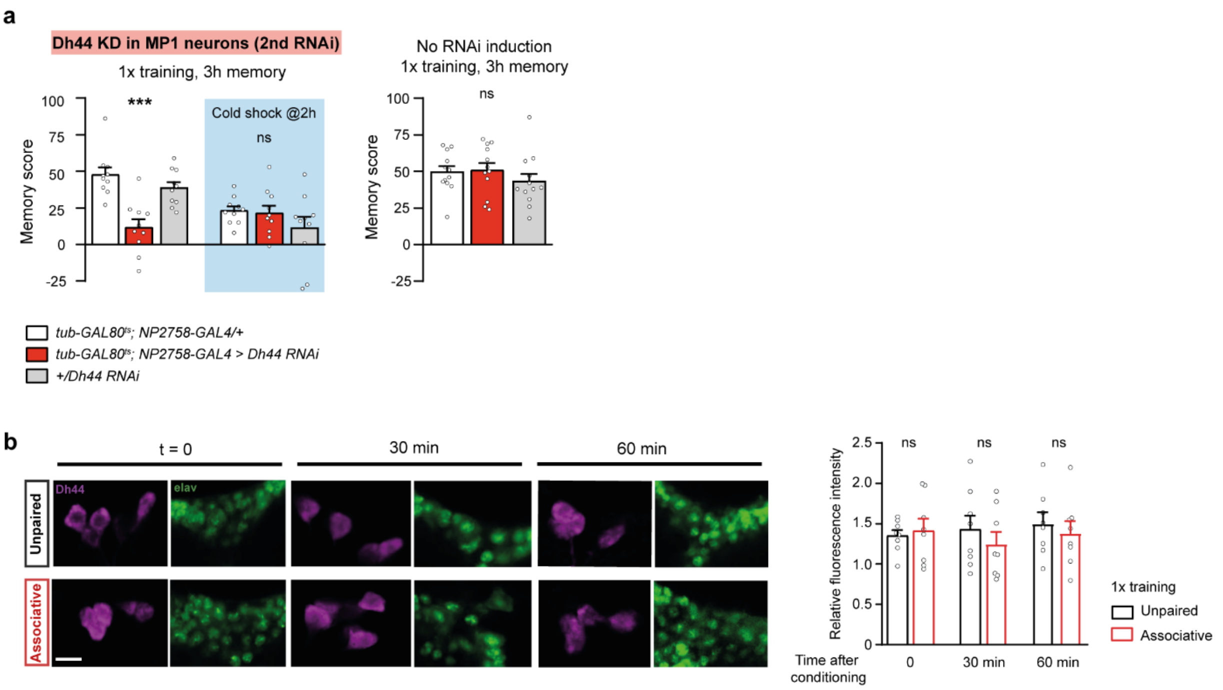

Supplementary Figure 3: Additional experiments confirming the specific need of acute release of Dh44 from MP1 neurons for memory.

(**a**) *Dh44* KD in MP1 neurons using a second non-overlapping RNAi targeting *Dh44* impaired total memory performance after single-cycle training (n = 10, $F_{2,27}$ = 14.69, p = 0.00004) but did not affect cold shock-resistant memory (n = 10, $F_{2,27}$ = 1.30, p = 0.28). When RNAi expression was not induced, flies showed normal memory after single-cycle training (n = 12, $F_{2,33}$ = 0.74, p = 0.48).

(**b**) Immunostaining time series showing Dh44 peptide in Dh44 neurons (with elav as neuronal counterstaining) in the PI at different time points after single-cycle training or unpaired conditioning protocol. No difference in relative fluorescence intensity was detected at any time point (n = 8 for all timepoints; t = 0 min: t_{14} = 0.41, p = 0.68; t = 30 min: t_{14} = 0.78, p = 0.44; t = 60 min: t_{14} = 0.57, p = 0.57). Scale bar: 10 µm.

RNAi line KK110160 (*Dh44*) was used in this figure. Data are represented as mean ± SEM. ns: not significant, p > 0.05, ***p < 0.001 by two-tailed Student's t test (panel b) or Tukey's pairwise comparison following one-way ANOVA (panel a). Source data are provided as a Source Data file.

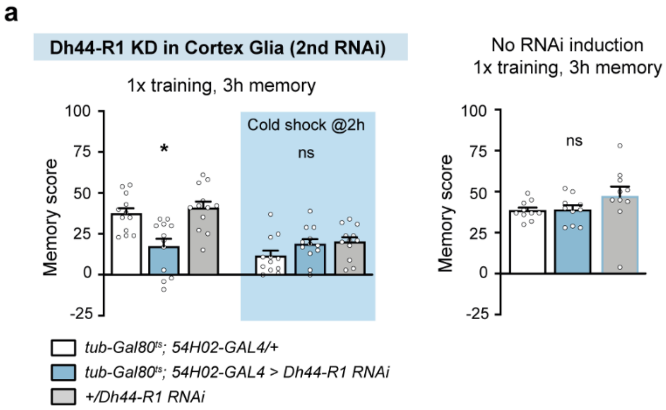

Supplementary Figure 4: Effect of Dh44-R1 knockdown in cortex glia on memory assayed with a second RNAi.

(**a**) *Dh44-R1* KD in cortex glia using a second non-overlapping RNAi targeting *Dh44-R1* impaired total memory performance after single-cycle training (n = 12, $F_{2,33} = 9.76$, $p = 0.0005$) but did not affect cold shock-resistant memory (n = 12, $F_{2,33} = 2.18$, $p = 0.13$). When RNAi expression was not induced, flies showed normal memory after single-cycle training (n = 10, $F_{2,27} = 1.56$, $p = 0.22$).

RNAi line JF03208 (*Dh44-R1*) was used in this figure. Data are represented as mean ± SEM. ns: not significant, $p > 0.05$, $*p < 0.05$ by Tukey's pairwise comparison following one-way ANOVA. Source data are provided as a Source Data file.

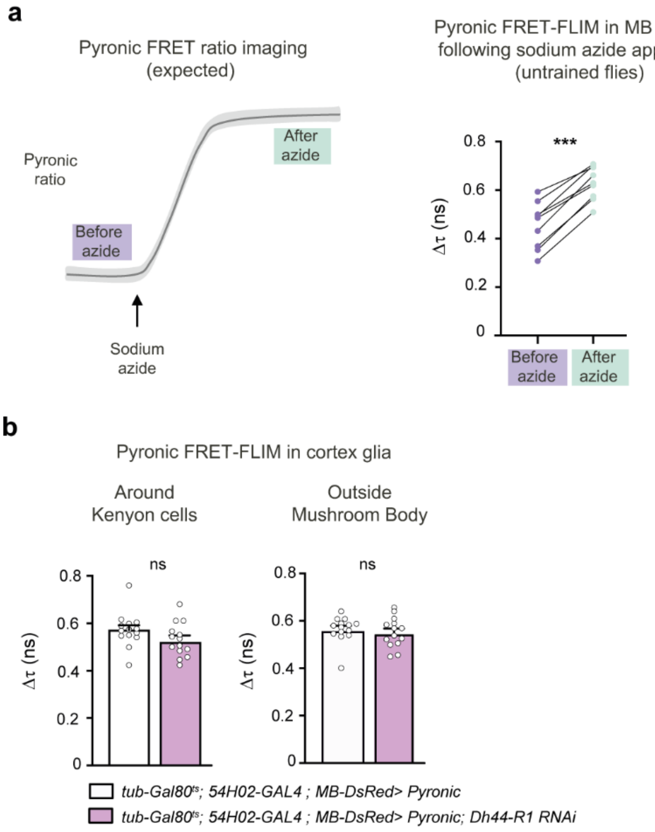

Supplementary Figure 5: **Validation experiments for the measurement of cellular pyruvate levels by FRET-FLIM of the Pyronic sensor.**

(**a**) Validation of the measurement of the donor fluorophore lifetime (FRET-FLIM) as a reporter of pyruvate level. When expressed in KCs (VT30559-GAL4 > UAS-Pyronic), the inverted FRET ratio of the Pyronic sensor (mTFP/Venus intensities) reports a robust and fast increase in pyruvate following azide application (5 mM) (as reported in Figure 3; the time trace on this panel is an illustration and does not correspond to actual experimental data). As expected, FRET-FLIM measurement in KC axons showed a strong increase in mTFP lifetime (see Methods) due to azide treatment (n = 9, t_{16} = 4.43, p = 0.0004). As per the design of the Pyronic sensor, increased pyruvate idecreases FRET between mTFP and Venus, which is expected to increase the measured mTFP lifetime.

(**b**) FRET-FLIM of Pyronic sensor expressed in cortex glia in naive flies. No significant difference in cortex glia pyruvate levels was observed between control flies and flies where *Dh44-R1* was KD in cortex glia around KCs (n = 14, t_{26} = 1.62, p = 0.11). Likewise, no difference between the groups was observed in a cortex glia region outside MB (as illustrated on Figure 4; n = 14, t_{26} = 0.60, p = 0.54). RNAi line JF03208 (*Dh44-R1*) was used in this figure. Data are represented as mean ± SEM. ns: not

significant, $p > 0.05$, $*p < 0.05$ by two-tailed Student's t test. Source data are provided as a Source Data file.

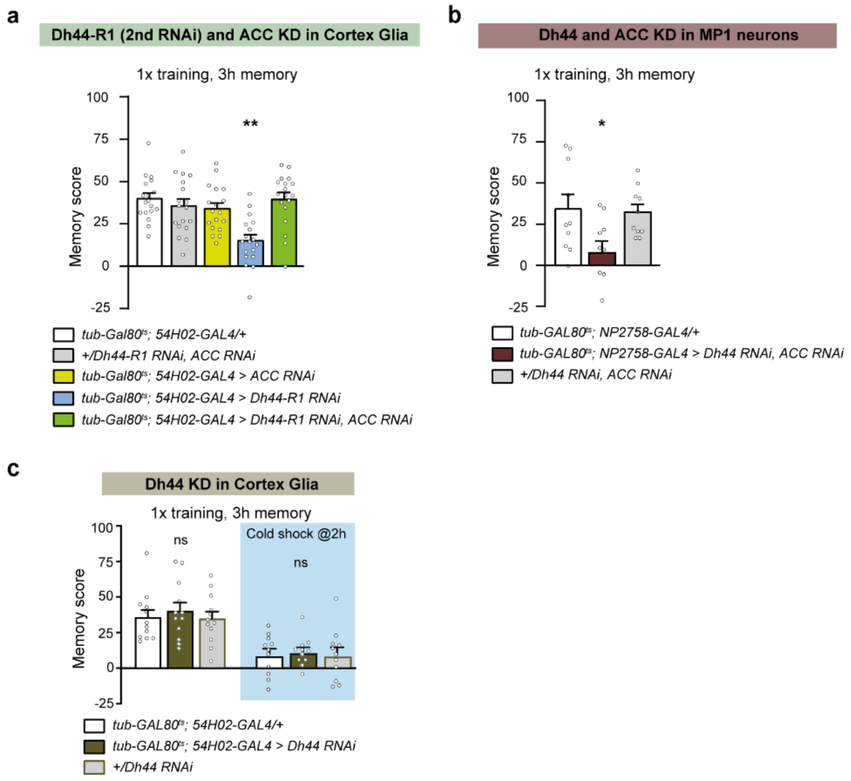

Supplementary Figure 6: Additional experiments confirming the rescue by ACC knockdown of memory impairments caused by defective Dh44 signalling.

(**a**) Dual *Dh44-R1* and *ACC* KD in cortex glia, using the second *Dh44-R1* RNAi, rescues the memory deficit observed upon the single KD of *Dh44-R1* (n = 18, $F_{4,55}$ = 14.53, p = 6.10^{-6}).

(**b**) Memory impairment after single-cycle training could still be observed when the dual *Dh44* and *ACC* knockdown was induced in MP1 neurons only (n = 10, $F_{2,27}$ = 4.66, p = 0.018).

(**c**) *Dh44* KD in cortex glia did not affect memory performance after single-cycle training (n = 12, $F_{2,33}$ = 0.29, p = 0.74) and cold shock-resistant memory was normal (n = 11, $F_{2,30}$ =0.08, p = 0.91).

RNAi lines JF03208 (*Dh44-R1*), GD3482 (*ACC*) and JF01822 (*Dh44*) were used in this figure. Data are represented as mean ± SEM. ns: not significant, p > 0.05, *p < 0.05, **p < 0.01 by Tukey's pairwise comparison following one-way ANOVA. Source data are provided as a Source Data file.

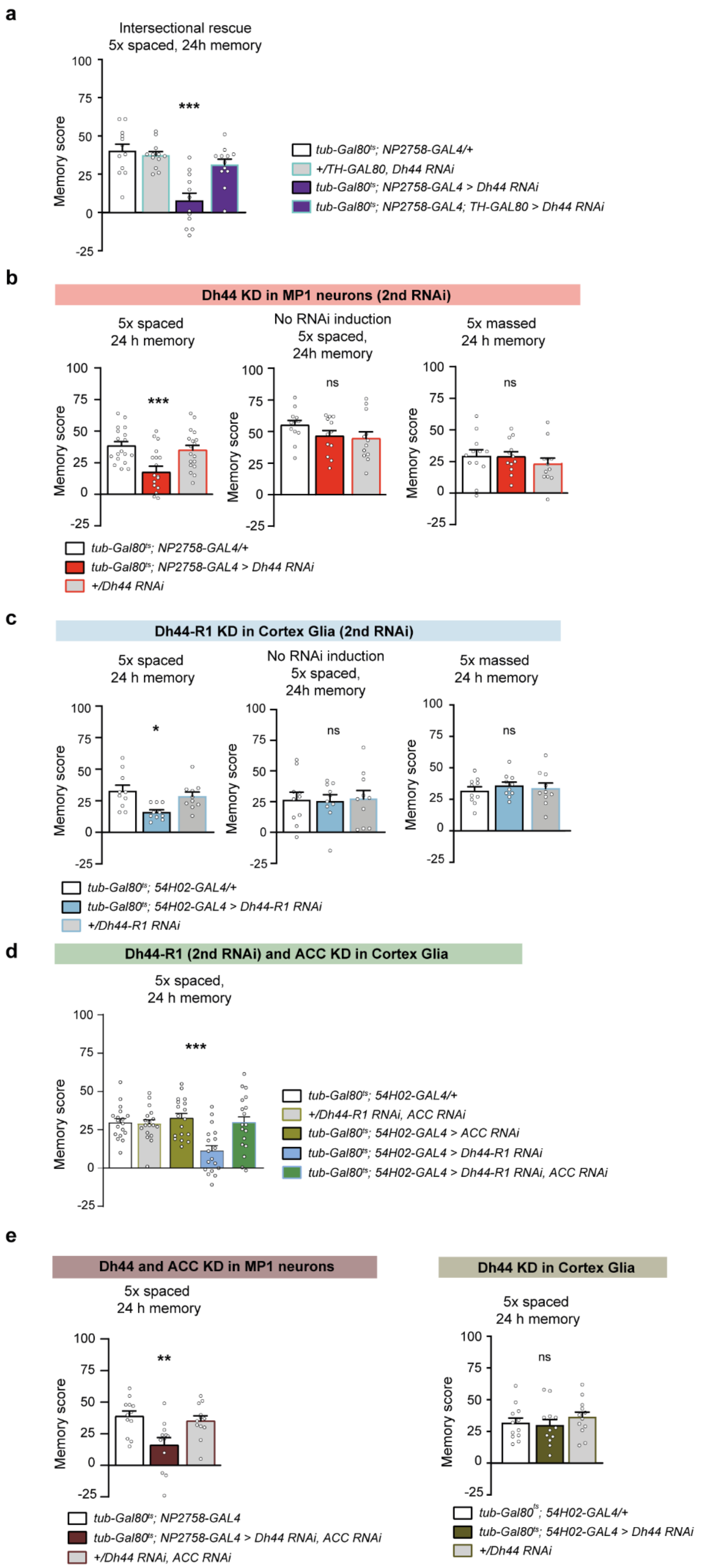
a
Intersectional rescue
5x spaced, 24h memory
Memory score
***
tub-Gal80^{ts}; NP2758-GAL4/+
+/TH-GAL80, Dh44 RNAi
tub-Gal80^{ts}; NP2758-GAL4 > Dh44 RNAi
tub-Gal80^{ts}; NP2758-GAL4; TH-GAL80 > Dh44 RNAi
b
Dh44 KD in MP1 neurons (2nd RNAi)
5x spaced
24 h memory
No RNAi induction
5x spaced,
24h memory
5x massed
24 h memory
Memory score
***
ns
ns
tub-Gal80^{ts}; NP2758-GAL4/+
tub-Gal80^{ts}; NP2758-GAL4 > Dh44 RNAi
+/Dh44 RNAi
c
Dh44-R1 KD in Cortex Glia (2nd RNAi)
5x spaced
24 h memory
No RNAi induction
5x spaced,
24h memory
5x massed
24 h memory
Memory score
*
ns
ns
tub-Gal80^{ts}; 54H02-GAL4/+
tub-Gal80^{ts}; 54H02-GAL4 > Dh44-R1 RNAi
+/Dh44-R1 RNAi
d
Dh44-R1 (2nd RNAi) and ACC KD in Cortex Glia
5x spaced,
24 h memory
Memory score
***
tub-Gal80^{ts}; 54H02-GAL4/+
+/Dh44-R1 RNAi, ACC RNAi
tub-Gal80^{ts}; 54H02-GAL4 > ACC RNAi
tub-Gal80^{ts}; 54H02-GAL4 > Dh44-R1 RNAi
tub-Gal80^{ts}; 54H02-GAL4 > Dh44-R1 RNAi, ACC RNAi
e
Dh44 and ACC KD in MP1 neurons
5x spaced
24 h memory
Memory score
**
tub-Gal80^{ts}; NP2758-GAL4
tub-Gal80^{ts}; NP2758-GAL4 > Dh44 RNAi, ACC RNAi
+/Dh44 RNAi, ACC RNAi
Dh44 KD in Cortex Glia
5x spaced
24 h memory
Memory score
ns
tub-Gal80^{ts}; 54H02-GAL4/+
tub-Gal80^{ts}; 54H02-GAL4 > Dh44 RNAi
+/Dh44 RNAi

Supplementary Figure 7: Additional experiments demonstrating the role of Dh44 signalling in LTM.

(**a**) Inhibition of GAL4 expression only in MP1 neurons by using the *TH-GAL80* transgene failed to induce an LTM defect (n = 12, $F_{3,44}$ = 13.53, p = 2.10^{-6}).

(**b**) *Dh44* KD in MP1 neurons, using a second non-overlapping RNAi targeting *Dh44*, impaired LTM after spaced training (n = 18, $F_{2,51}$ = 8.10, p = 0.0009) but not memory resulting from massed training (n = 12, $F_{2,33}$ = 0.57, p = 0.56). When RNAi expression was not induced, flies showed normal LTM after spaced training (n = 12, $F_{2,33}$ = 1.51, p = 0.23).

(**c**) *Dh44-R1* KD in cortex glia, using a second non-overlapping RNAi targeting *Dh44-R1*, impaired LTM after spaced training (n = 10, $F_{2,27}$ = 6.21, p = 0.006) but not memory resulting from massed training (n = 10, $F_{2,27}$ = 0.02, p = 0.97). When RNAi expression was not induced, flies showed normal LTM after spaced training (n = 10, $F_{2,27}$ = 0.32, p = 0.72).

(**d**) Dual *Dh44-R1* and *ACC* knockdown (KD) in cortex glia, using the second *Dh44-R1* RNAi, rescued the LTM deficit after spaced training observed upon the single KD of *Dh44-R1* (n = 18, $F_{4,55}$ = 8.73, p = 6.10^{-5}).

(**e**) LTM impairment after spaced training could still be observed when the dual *Dh44* and *ACC* knockdown was induced in MP1 neurons only (n = 12, $F_{2,33}$ = 6.71, p = 0.0036). *Dh44* KD in cortex glia did not affect LTM performance (n = 12, $F_{2,33}$ = 0.58, p = 0.56). RNAi lines JF01822 (*Dh44*, panel a, e), KK110160 (*Dh44*, panel b), JF03208 (*Dh44-R1*) and GD3482 (*ACC*) were used in this figure. Data are expressed as mean ± SEM. ns: not significant, $p > 0.05$, *$p < 0.05$, **$p < 0.01$, ***$p < 0.001$ by Tukey's pairwise comparison following one-way ANOVA. Source data are provided as a Source Data file.

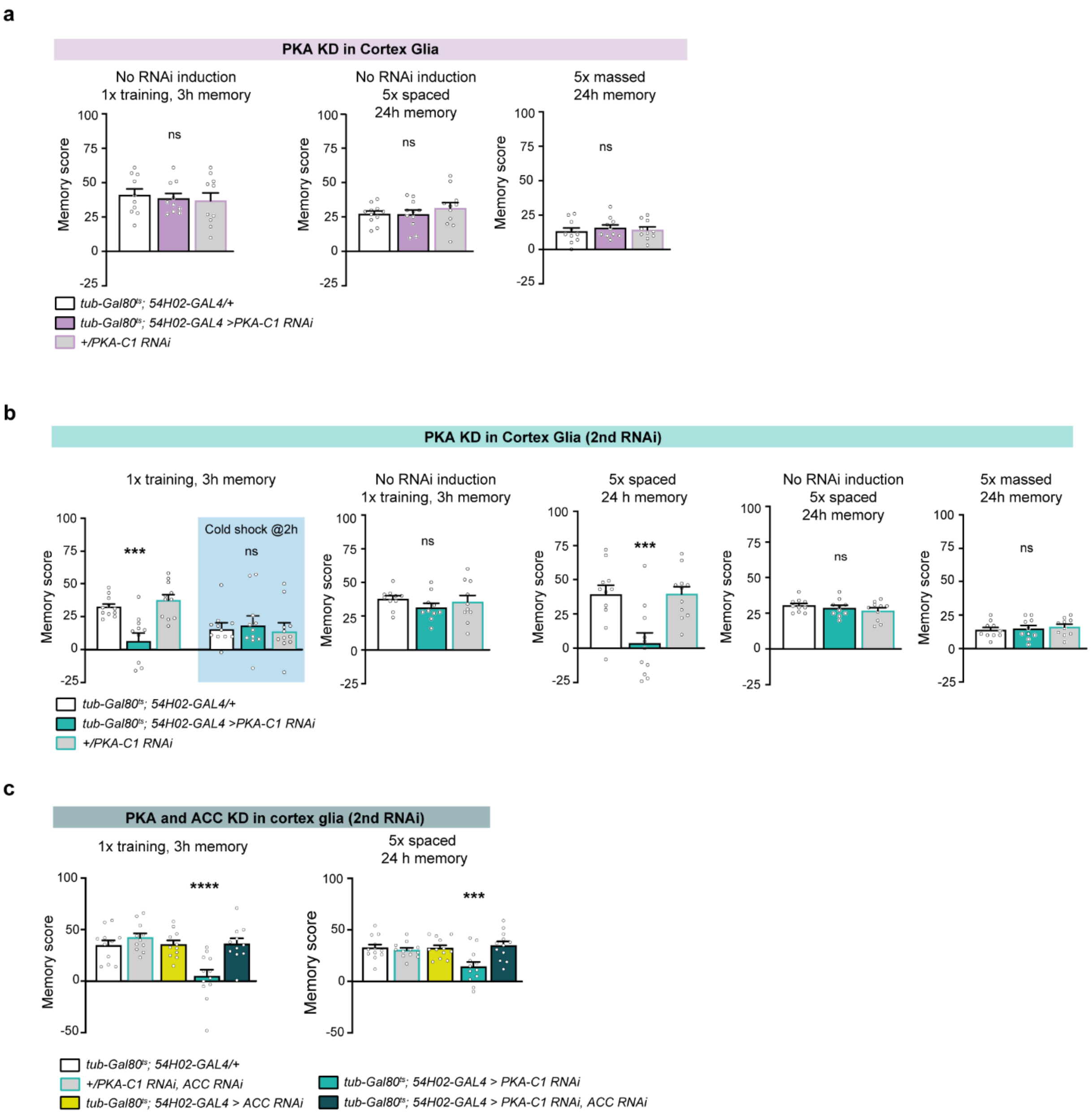

Supplementary Figure 8: Additional experiments supporting a role of PKA in cortex glia downstream Dh44 signalling for memory formation.

(**a**) When RNAi expression was not induced, flies showed normal memory after single-cycle training (n = 10, $F_{2,27}$ = 0.18, p = 0.83). and normal LTM after spaced training (n = 11, $F_{2,30}$ = 0.55, p = 0.58). RNAi-based knockdown of PKA-C1 in cortex glia did not affect memory after massed training (n = 10, $F_{2,27}$ = 0.28, p = 0.75).

(**b**) *PKA-C1* KD in cortex glia, using a second non-overlapping RNAi targeting *PKA-C1*, impaired MTM after single-cycle training (n = 11, $F_{2,30}$ = 13,68, p = 0.00006). When RNAi expression was not induced, flies showed normal memory after single-cycle training (n = 10, $F_{2,27}$ = 0.79, p = 0.46). LTM impairment

was also observed using this second non-overlapping RNAi targeting *PKA-C1* in cortex glia (n = 11, $F_{2,30}$ = 9.36, p = 0.0007) while normal LTM was observed when the RNAi expression was not induced (n = 10, $F_{2,27}$ = 0.99, p = 0.38). No memory defect was observed after massed training (n = 10, $F_{2,27}$ = 0.26, p = 0.76).

(**c**) The dual *PKA-C1* and *ACC* KD in cortex glia rescued the memory measured 3 hrs after single-cycle training (n = 11;11;11;12;11 from left to right, $F_{4,50}$ = 5,81, p = 0.0006) and LTM deficit after spaced training observed upon the single KD of *PKA-C1* (n = 12, $F_{4,55}$ = 5.55, p = 0.0008).

RNAi lines JF01188 (*PKA-C1* panel a), JF01218 (*PKA-C1*, panel b, c) and GD3482 (*ACC*, panel c) were used in this figure. Data are expressed as means ± SEM. ns: not significant, $p > 0.05$, *$p < 0.05$, **$p < 0.01$, ***$p < 0.001$ by Tukey's pairwise comparison following one-way ANOVA. Source data are provided as a Source Data file.

Supplementary Table 1: Innate odour and electric shock avoidance in flies with *Dh44* knockdown in MP1 neurons. Data are represented as mean ± SEM. Statistics were derived by one-way ANOVA followed by Tukey's pairwise comparison. Source data are provided as a Source Data file.

Genotypes	Shock Avoidance		Naive odour avoidance			
			Octanol		Methylcyclohexanol	
	Mean± S.E.M	Statistics	Mean± S.E.M	Statistics	Mean± S.E.M	Statistics
Tub-Gal80^{ts};NP2758-GAL4/+	48.9 ± 6.3	$F_{2,33} = 0.13$ p = 0.87 n = 12	60.7 ± 5.2	$F_{2,27} = 3.28$ p = 0.17 n = 10	48.2 ± 3.7	$F_{2,27} = 1.43$ p = 0.25 n = 10
Tub-Gal80^{ts}; NP2758-GAL4> UAS-Dh44$^{RNAi\ JF01822}$	51.0 ± 6.2		56.4 ± 5.0		61.2 ± 5.4	
UAS-Dh44$^{RNAi\ JF01822}$/+	47.0 ± 2.9		48.3 ± 3.7		54.5 ± 6.7	
Tub-Gal80^{ts};NP2758-GAL4/+	48.2 ± 4.9	$F_{2,33} = 0.31$ p = 0.72 n = 12	49.9 ± 3.3	$F_{2,32} = 0.60$ p = 0.55 n = 12	57.2 ± 4.2	$F_{2,32} = 0.05$ p = 0.94 n = 12
Tub-Gal80^{ts}; NP2758-GAL4> UAS-Dh44$^{RNAi\ KK110160}$	48.6 ± 3.1		53.0 ± 4.4		55.2 ± 6.5	
UAS-Dh44$^{RNAi\ KK110160}$/+	44.6 ± 3.3		56.4 ± 4.7		57.3 ± 4.2	

Supplementary Table 2: Innate odour and electric shock avoidance in flies with *Dh44-R1* knockdown in cortex glia. Data are represented as mean ± SEM. Statistics were derived by one-way ANOVA followed by Tukey's pairwise comparison. Source data are provided as a Source Data file.

Genotypes	Shock Avoidance		Naive odour avoidance			
			Octanol		Methylcyclohexanol	
	Mean± S.E.M	Statistics	Mean± S.E.M	Statistics	Mean± S.E.M	Statistics
Tub-Gal80^{ts};54H02-GAL4/+	69.7 ± 6.3	$F_{2,33}$ = 0.08 p = 0.92 n = 12	70.5 ± 5.8	$F_{2,27}$ = 1.91 p = 0.17 n = 10	45.4 ± 7.1	$F_{2,27}$ = 0.23 p = 0.79 n = 10
Tub-Gal80^{ts}; 54H02-GAL4> UAS-Dh44-R1$^{RNAi\ KK108591}$	66.2 ± 6.3		57.0 ± 5.1		50.6 ± 5.1	
UAS-Dh44-R1$^{RNAi\ KK108591}$/+	69.9 ± 9.0		56.8 ± 6.0		48.0 ± 3.1	
Tub-Gal80^{ts}; 54H02-GAL4/+	40.2 ± 1.9	$F_{2,27}$ = 0.33 p = 0.72 n = 10	50.1 ± 3.0	$F_{2,27}$ = 0.95 p = 0.55 n = 10	58.2 ± 3.8	$F_{2,27}$ = 1.30 p = 0.28 n = 10
Tub-Gal80^{ts}; 54H02-GAL4> UAS-Dh44$^{RNAi\ JF03208}$	43.5 ± 4.3		51.6 ± 3.7		50.7 ± 4.9	
UAS-Dh44$^{RNAi\ JF03208}$/+	41.7 ±1.5		51.6 ± 3.0		59.6 ±3.7	

Supplementary Table 3: Validation of the *PKA-C1* RNAi constructs by qRT-PCR. Data are represented as mean ± SEM. Statistics were derived by two-tailed Student's t test.

Genotypes	Mean ± SEM	Statistics	% of mRNA reduction
Repo-GAL4/+	0.57 ± 0.03	n = 4<br>t = 4.36<br>p = 0.004	35%
Repo-GAL4> UAS-PKA-C1 $^{RNAi\ JF\ 01218}$	0.37 ± 0.02		
Repo-GAL4/+	1.30 ± 0.35	n = 6<br>t = 2.85<br>p = 0.01	80%
Repo-GAL4>UAS-PKA-C1 $^{RNAi\ JF\ 01188}$	0.26 ± 0.07		

Supplementary Table 4: Innate odour and electric shock avoidance in flies with *PKA-C1* knockdown in cortex glia. Data are represented as mean ± SEM. Statistics were derived by one-way ANOVA followed by Tukey's pairwise comparison. Source data are provided as a Source Data file.

Genotypes	Shock Avoidance		Naive odour avoidance			
			Octanol		Methylcyclohexanol	
	Mean± S.E.M	Statistics	Mean± S.E.M	Statistics	Mean± S.E.M	Statistics
Tub-Gal80^{ts}*;54H02-GAL4/+*	54.4 ± 5.6	$F_{2,27}$ = 0.15<br>p = 0.86<br>n = 10	52.5 ± 5.7	$F_{2,27}$ = 0.76<br>P = 0.47<br>n = 10	56.3 ± 5.4	$F_{2,27}$ = 0.18<br>p = 0.83<br>n = 10
Tub-Gal80^{ts}*; 54H02-GAL4> UAS-PKA-C1* ^{RNAiJF01188}	53.2 ± 3.6		57.6 ± 3.0		52.4 ± 3.6	
UAS-PKA-C1 ^{RNAiJF01188}*/+*	50.8 ± 4.7		49.0 ± 5.6		53.8 ± 4.7	
Tub-Gal80^{ts}*;54H02-GAL4/+*	50.6 ± 5.8	$F_{2,27}$=0.26<br>p = 0.76<br>n = 10	55.8 ± 4.6	$F_{2,27}$ = 0.74<br>p = 0.48<br>n = 10	53.6 ± 4.0	$F_{2,27}$ = 0.64<br>p = 0.53<br>n = 10
TubGal80^{ts}*; 54H02-GAL4> UAS-PKA-C1* ^{RNAiJF01218}	48.6 ± 4.6		48.8 ± 5.9		46.5 ± 5.5	
UAS-PKA-C1 ^{RNAiJF01218}*/+*	45.6 ± 3.9		46.5 ± 6.2		49.4 ± 3.6	

Supplementary Note 1: comparison of ACC expression between cortex glia and MP1 neurons using publicly available single-cell transcritpomics datasets.

Using the single-cell RNAseq dataset from Aso et al. (2019) (ref. [5]), we extracted the transcriptome of two neurons labeled as PPL1γ1-pedc (aka MP1 neurons). Both cells give quite consistent ranking of tyrosine hydroxylase (ple), Dh44, and ACC. In the first cell, out of ~9600 non-zero measured transcripts, TH ranks #12, Dh44 ranks #98 (~600 counts) and ACC ranks #5622 (ACC-RF :~3 counts). In the second one, out of ~18000 non-zero measured transcripts, TH ranks #136, Dh44 ranks #368 (~110 counts) and ACC ranks #6039 (ACC-RF :~5 counts). Thus, cell-wise, ACC expression is very low compared to Dh44, or to TH, another gene well-known to be functionally relevant for these neurons.

Unfortunately, this dataset does not include cortex glia for direct comparison of ACC levels with MP1 neurons within the same experimental conditions. On the other hand, it is not straightforward to identify unequivocally MP1 neurons in larger datasets such as the one from Davie et al. (2018) (ref. [6]). Therefore, to achieve a reliable comparison between MP1 neurons and cortex glia, we used two distinct datasets (Aso et al., 2018 ; Davie et al., 2018), using α/β Kenyon cells as the common reference since they are present in the study by Aso et al., and are easily identifiable in the dataset published by Davie et al. According to Aso et al., like in MP1 neurons, ACC-RF is the highly expressed ACC transcript in Kenyon cells. In the three α/β Kenyon cells present in the dataset, ACC-RF counts are 17.24 ; 11.79 ; 22.68 respectively, i.e ~17 on average , more than 4 times higher than the ~4 counts measured in MP1 neurons. Turning to the Davie et al. dataset, qualitative examination using the Scope web interface

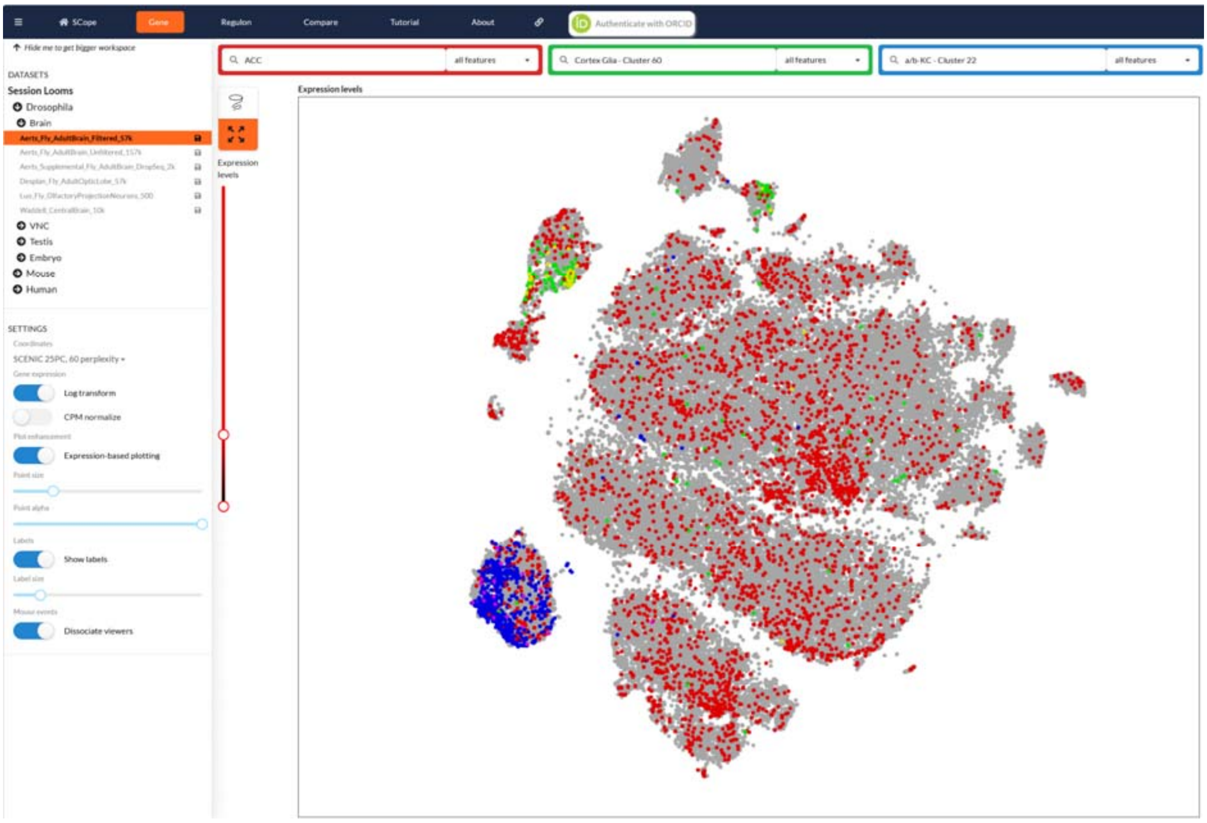

(https://scope.aertslab.org/#/Davie_et_al_Cell_2018/Davie_et_al_Cell_2018%2FAerts_Fly_AdultBrain_Filtered_57k.loom/gene) reveals expression scattered in many cell types, either glial or neuronal.

However, a more quantitative analysis of this dataset reveals on average a much higher transcript detection in cells of the cortex glia cluster (0.20 ± 0.03 (mean ± SEM), including 16% cells with non-zero detection) than in the α/β Kenyon cells cluster (0.029 ± 0.006 (mean ± SEM), including 3% cells with non-zero detection).

In conclusion, the co-consideration of both datasets is supportive of the fact that ACC expression in MP1 neurons is much lower than in cortex glia.

Comparison of Dh44 expression between cortex glia and MP1 neurons using publicly available single-cell transcriptomics datasets.

Using the Scope web interface to visualize the data from Davie et al. (2018), it is clear that brain cells expressing Dh44 and cells belonging to the cortex glia cluster do not overlap.

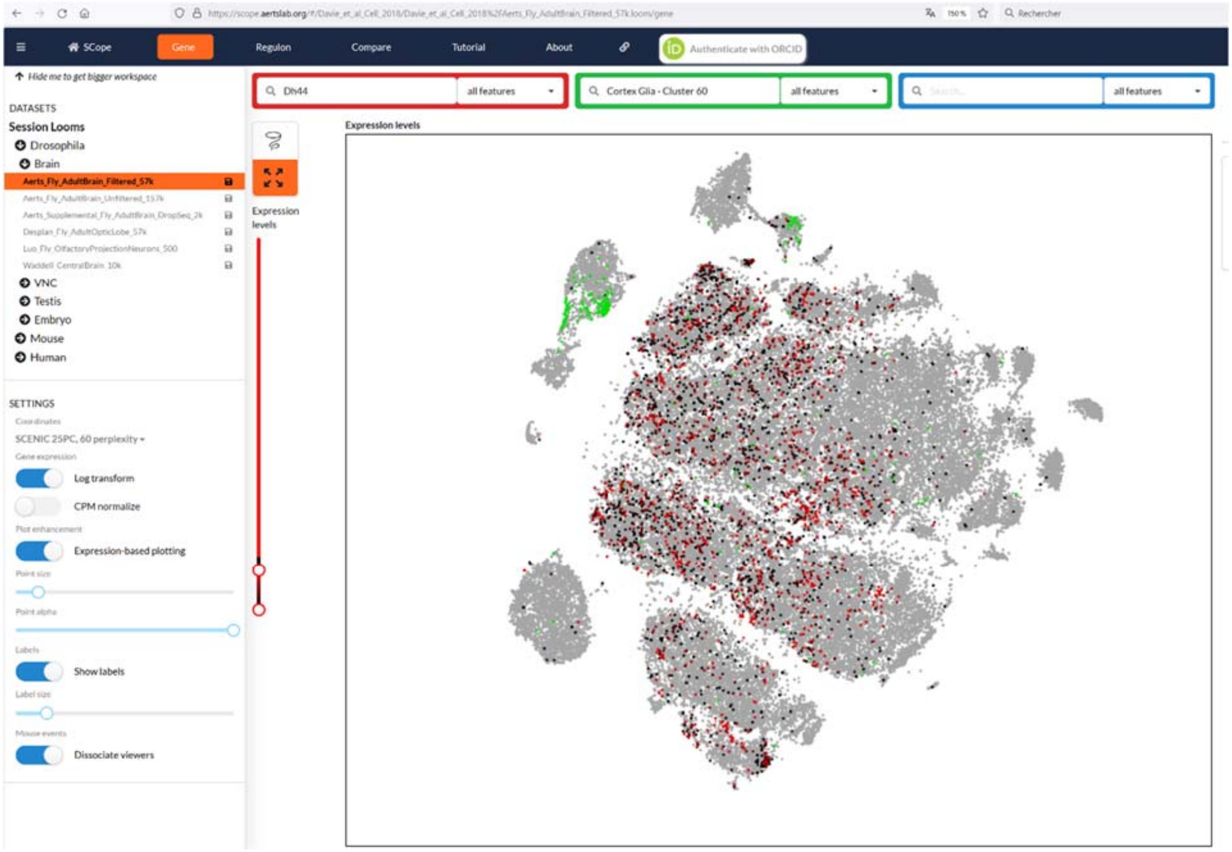

Supplementary Note 2 :Comparisons of rat and Drosophila ACC

PBlast alignment of rat ACC (Query) and Drosophila melanogaster ACC isoform ACC-A. Conserved serines in position 77, 79 and 1200 (rat ACC) are highlighted in yellow. Of note, all drosophila ACC isoforms share the same aminoacid sequence as ACC-A for the first 10 exons, which include the relevant sites.

Query: SP|P11497|ACACA_RAT ACETYL-COA CARBOXYLASE 1 OS=RATTUS NORVEGICUS OX=10116 GN=ACACA PE=1 SV=1 Query ID: lcl|Query_3659723 Length: 2345

>tr|A1Z784|A1Z784_DROME Acetyl-CoA carboxylase, isoform A OS=Drosophila melanogaster OX=7227 GN=ACC PE=1 SV=1

Sequence ID: Query_3659725 Length: 2482

Range 1: 222 to 2470

Score:2917 bits(7561), Expect:0.0,

Method:Compositional matrix adjust.,

Identities:1426/2296(62%), Positives:1768/2296(77%), Gaps:76/2296(3%)

```
Query  74    MRSSMS-GLHLVKQGRDRKKIDSQRDFTVASPAEFVTRFGGNKVIEKVLIANNGIAAVKC  132
             +R SMS G  L   G+DR +    RDF +A+  EFV RFGG +VI KVLIANNGIAAVKC
Sbjct  222   LRPSMSRGTGL---GQDRHQ---DRDFHIATTEEFVKRFGGTRVINKVLIANNGIAAVKC  275

Query  133   MRSIRRWSYEMFRNERAIRFVVMVTPEDLKANAEYIKMADHYVPVPGGANNNNYANVELI  192
             MRSIRRW+YEMF+NERAIRFVVMVTPEDLKANAEYIKMADHYVPVPGG+NNNNYANVELI
Sbjct  276   MRSIRRWAYEMFKNERAIRFVVMVTPEDLKANAEYIKMADHYVPVPGGSNNNNYANVELI  335

Query  193   LDIAKRIPVQAVWAGWGHASENPKLPELLLKNGIAFMGPPSQAMWALGDKIASSIVAQTA  252
             +DIA R  VQAVWAGWGHASENPKLPELL K G+ F+GPP +AMWALGDK+ASSIVAQTA
Sbjct  336   VDIALRTQVQAVWAGWGHASENPKLPELLHKEGLVFLGPPERAMWALGDKVASSIVAQTA  395

Query  253   GIPTLPWSGSGLRVDWQENDFSKRILNVPQDLYEKGYVKDVDDGLKAAEEVGYPVMIKAS  312
              IPTLPWSGS L+       +S + + +  +L+ +G V +V+ GL A  ++G+PVMIKAS
Sbjct  396   EIPTLPWSGSDLKAQ-----YSGKKIKISSELFARGCVTNVEQGLAAVNKIGFPVMIKAS  450
```

```
Query  313   EGGGGKGIRKVNNADDFPNLFRQVQAEVPGSPIFVMRLAKQSRHLEVQILADQYGNAISL  372
             EGGGGKGIR+V+  ++FP LFRQVQAEVPGSPIFVM+LA+ +RHLEVQ+LADQYGNAISL
Sbjct  451   EGGGGKGIRRVDTTEEFPGLFRQVQAEVPGSPIFVMKLARGARHLEVQLLADQYGNAISL  510

Query  373   FGRDCSVQRRHQKIIEEAPAAIATPAVFEHMEQCAVKLAKMVGYVSAGTVEYLYSQDGSF  432
             FGRDCS+QRRHQKIIEEAPA +A P VFE ME+ AV+LAKMVGYVSAGTVEYLY  +G +
Sbjct  511   FGRDCSIQRRHQKIIEEAPAIVAQPEVFEDMEKAAVRLAKMVGYVSAGTVEYLYDPEGRY  570

Query  433   YFLELNPRLQVEHPCTEMVADVNLPAAQLQIAMGIPLFRIKDIRMMYGVSPWGDAPIDFE  492
             +FLELNPRLQVEHPCTEMVADVNLPAAQLQI MGIPL+R+KDIR++YG SPWG + IDFE
Sbjct  571   FFLELNPRLQVEHPCTEMVADVNLPAAQLQIGMGIPLYRLKDIRLLYGESPWGSSVIDFE  630

Query  493   NSAHVPCPRGHVIAARITSENPDEGFKPSSGTVQELNFRSNKNVWGYFSVAAAGGLHEFA  552
             N  + P P GHVIAARITSENPDEGFKPSSGTVQELNFRS+KNVWGYFSVAA+GGLHEFA
Sbjct  631   NPPNKPRPSGHVIAARITSENPDEGFKPSSGTVQELNFRSSKNVWGYFSVAASGGLHEFA  690

Query  553   DSQFGHCFSWGENREEAISNMVVALKELSIRGDFRTTVEYLIKLLETESFQLNRIDTGWL  612
             DSQFGHCFSWGENR++A  N+V+ALKELSIRGDFRTTVEYLI LLET  F  N IDT WL
Sbjct  691   DSQFGHCFSWGENRQQARENLVIALKELSIRGDFRTTVEYLITLLETNRFLDNSIDTAWL  750

Query  613   DRLIAEKVQAERPDTMLGVVCGALHVADVNLRNSISNFLHSLERGQVLPAHTLLNTVDVE  672
             D LIAE+VQ+E+PD +LGV+CG+LH+AD  +  S S+F  SLE+GQ+  A+TL N VDVE
Sbjct  751   DALIAERVQSEKPDILLGVMCGSLHIADRQITESFSSFQTSLEKGQIQAANTLTNVVDVE  810

Query  673   LIYEGIKYVLKVTRQSPNSYVVIMNGSCVEVDVHRLSDGGLLLSYDGSSYTTYMKEEVDR  732
             LI +GI+Y ++  +   NSY ++MN S  E++VHRLSDGGLL+S +G+SYTTYMKEEVDR
Sbjct  811   LINDGIRYKVQAAKSGANSYFLLMNSSFKEIEVHRLSDGGLLISLEGASYTTYMKEEVDR  870

Query  733   YRITIGNKTCVFEKENDPSVMRSPSAGKLIQYIVEDGGHVFAGQCYAEIEVMKMVMTLTA  792
             YRI IGN+TCVFEKENDPS++RSPSAGKLI  IVEDG HV  GQ YAEIEVMKMVMTLT+
Sbjct  871   YRIVIGNQTCVFEKENDPSLLRSPSAGKLINMIVEDGAHVSKGQAYAEIEVMKMVMTLTS  930

Query  793   VESGCIHYVKRPGAALDPGCVIAKMQLDNPSKVQQAELHTGSLPQIQSTALRGEKLHRVF  852
```

```
              E+G + +V+RPGA LD G ++  ++LD+PS V +A+   G   Q ++  +  EKL+RV
Sbjct  931   QEAGTVTFVRRPGAVLDAGSLLGHLELDDPSLVTKAQPFKGQFLQPENAPV-PEKLNRVH  989

Query  853   HYVLDNLVNVMNGYCLPDPFFSSKVKDWVERLMKTLRDPSLPLLELQDIMTSVSGRIPLN  912
             +     L N + GYCLP+PF + +++D +E+ M++LRDPSLPLLELQ+++ S+SGRIP++
Sbjct  990   NTYKSILENTLAGYCLPEPFNAQRLRDIIEKFMQSLRDPSLPLLELQEVIASISGRIPIS  1049

Query  913   VEKSIKKEMAQYASNITSVLCQFPSQQIANILDSHAATLNRKSEREVFFMNTQSIVQLVQ  972
             VEK I+K M  Y  NITSVL QFPSQQIA+++DSHAATL ++++R+VFF+ TQSIVQLVQ
Sbjct  1050  VEKKIRKLMTLYERNITSVLAQFPSQQIASVIDSHAATLQKRADRDVFFLTTQSIVQLVQ  1109

Query  973   RYRSGIRGHMKAVVMDLLRQYLRVETQFQNGHYDKCVFALREENKSDMNTVLNYIFSHAQ  1032
             RYR+GIRG MKA V +LLRQY  VE+QFQ GHYDKCV  +RE NK DM TV+N IFSH+Q
Sbjct  1110  RYRNGIRGRMKAAVHELLRQYYDVESQFQYGHYDKCVGLVREHNKDDMQTVVNTIFSHSQ  1169

Query  1033  VTKKNLLVTMLIDQLCGRDPTLTDELLNILTELTQLSKTTNAKVALRARQVLIASHLPSY  1092
             V KKNLLVT+LID L   +P LTDEL N L+ELT L++  +++VALR+RQVLIA+H P+Y
Sbjct  1170  VAKKNLLVTLLIDHLWANEPGLTDELANTLSELTSLNRAEHSRVALRSRQVLIAAHQPAY  1229

Query  1093  DVRHNQVESIFLSAIDMYGHQFCIENLQKLILSETSIFDVLPNFFYHSNQVVRMAALEVY  1152
             ++RHNQ+ESIFLSA+DMYGH F  ENLQ+LILSETSIFD+L +FFYHSN+ V  AALEVY
Sbjct  1230  ELRHNQMESIFLSAVDMYGHDFHPENLQRLILSETSIFDILHDFFYHSNRAVCNAALEVY  1289

Query  1153  VRRAYIAYELNSVQHRQLKDNTCVVEFQFMLPTSHPNRGNIPTLNRMSFASNLNHYGMTH  1212
             VRRAY +YEL  +QH +L     +V FQF+LPT+HPNR      +RMS        G+
Sbjct  1290  VRRAYTSYELTCLQHLELSGGLPLVHFQFLLPTAHPNR----LFSRMSSPD-----GLDQ  1340

Query  1213  VASVSDVLLDNAFTPPCQRMGGMVSFRTFEDFVRIFDEVMGCFCD--SPPQ---------  1261
              A+ S   L N+F     R G + +F +FE F    DE++    D  SP
Sbjct  1341  AAAES---LGNSFV----RTGAIAAFDSFEHFEMYSDEILDLLEDFVSPAMVNAKVLEAV  1393

Query  1262  --SPTFPESGHTSLYD---EDKVPR---------DEPIHILNVAIKTDGDIEDDRLAAMF  1307
               + +  +S H++  +    D V R          EPIHI++VA++  G+++D ++A +F
```

```
Sbjct  1394  EAADSISDSRHSTSINVSLSDPVTRANAAEEAKSTEPIHIVSVAVRETGELDDLQMAQIF  1453


Query  1308  REFTQQNKATLVEHGIRRLTFLVAQKDFRKQVNCEVDQRFHREFPKFFTFRARDKFEEDR  1367
               + Q++   L +  IRR+TF   +K               R+FPKFFTFRARDKF EDR
Sbjct  1454  GNYCQEHNEELFQRRIRRITFAALKK---------------RQFPKFFTFRARDKFTEDR  1498


Query  1368  IYRHLEPALAFQLELNRMRNFDLTAIPCANHKMHLYLGAAKVEVGTEVTDYRFFVRAIIR  1427
             IYRHLEPA AF LELNRM+ +DL A+P AN KMHLYLG AKV  G EVTDYRFF+R+IIR
Sbjct  1499  IYRHLEPASAFHLELNRMKTYDLEALPTANQKMHLYLGKAKVSKGQEVTDYRFFIRSIIR  1558


Query  1428  HSDLVTKEASFEYLQNEGERLLLEAMDELEVAFNNTNV-RTDCNHIFLNFVPTVIMDPSK  1486
             HSDL+TKEASFEYLQNEGER+LLEAMDELEVAF++ +  RTDCNHIFLNFVPTVIMDP+K
Sbjct  1559  HSDLITKEASFEYLQNEGERVLLEAMDELEVAFSHPHAKRTDCNHIFLNFVPTVIMDPAK  1618


Query  1487  IEESVRSMVMRYGSRLWKLRVLQAELKINIRLTTTGKAIPIRLFLTNESGYYLDISLYKE  1546
             IEESV  M+MRYG RLWKLRVLQAELK+ IR +       +RL + N+SGY+LDIS+Y E
Sbjct  1619  IEESVTKMIMRYGPRLWKLRVLQAELKMVIRQSPQSPTQAVRLCIANDSGYFLDISMYTE  1678


Query  1547  VTDSRTAQIMFQAYGDKQGPLHGMLINTPYVTKDLLQSKRFQAQSLGTTYIYDIPEMFRQ  1606
              T+  T  I F+AYG+KQG LHG  I+TPY+TKD LQ KRFQAQS GTTY+YD+P+MFRQ
Sbjct  1679  QTEPETGIIKFKAYGEKQGSLHGHPISTPYMTKDFLQQKRFQAQSNGTTYVYDVPDMFRQ  1738


Query  1607  SLIKLWESMSTQAFLPSPPLPSDILTYTELVLDDQGQLVHMNRLPGGNEIGMVAWKMSLK  1666
                + W   S          P  IL   + ++ +   LV M RLPG N  GMVAW++ L
Sbjct  1739  MTERHWREFSKARPTVDIRTPDKILIECKELVLEGDNLVEMQRLPGENNCGMVAWRIVLA  1798


Query  1667  SPEYPDGRDVIVIGNDITYRIGSFGPQEDLLFLRASELARAEGIPRIYVAANSGARIGLA  1726
             +PEYP+GR++IVI ND+TY IGSFG +ED+LF +AS+LAR   +PRIY++ NSGARIGLA
Sbjct  1799  TPEYPNGREIIVIANDLTYLIGSFGIKEDVLFAKASQLARQLKVPRIYISVNSGARIGLA  1858


Query  1727  EEIRHMFHVAWVDSEDPYKGYKYLYLTPQDYKRVSALNSVHCEHVEDEGESRYKITDIIG  1786
             EE++ MF +AW D E+P KG+KYLYL+ +DY +V+ LNSV    +EDEGE RYKITDIIG
Sbjct  1859  EEVKAMFKIAWEDPEEPDKGFKYLYLSTEDYAQVANLNSVRAILIEDEGEQRYKITDIIG  1918
```

```
Query  1787  KEEGLGAENLRGSGMIAGESSLAYDEIITISLVTCRAIGIGAYLVRLGQRTIQVENSHLI  1846
             K++GLG ENLR +G+IAGE+S AY+EI+TI++VTCR IGIG+Y+VRLGQR IQ++NSH+I
Sbjct  1919  KDDGLGVENLRYAGLIAGETSQAYEEIVTIAMVTCRTIGIGSYVVRLGQRVIQIDNSHII  1978

Query  1847  LTGAGALNKVLGREVYTSNNQLGGIQIMHNNGVTHCTVCDDFEGVFTVLHWLSYMPKNVH  1906
             LTG  ALNK+LGR+VY SNNQLGG QIM NNGVTH T   D +GV+T+L WLSY+P  +
Sbjct  1979  LTGYAALNKLLGRKVYASNNQLGGTQIMFNNGVTHKTEAIDLDGVYTILDWLSYIPAYIG  2038

Query  1907  SSVPLLNSKDPIDRIIEFVPTKAPYDPRWMLAGRPHPTQKGQWLSGFFDYGSFSEIMQPW  1966
               +P++   D I+R ++F+PTK+PYDPRWML GR +P     W +GFFD  S+SEIM  W
Sbjct  2039  CDLPIVLPNDRIERPVDFMPTKSPYDPRWMLGGRVNPVNANDWENGFFDRDSWSEIMASW  2098

Query  1967  AQTVVVGRARLGGIPVGVVAVETRTVELSVPADPANLDSEAKIIQQAGQVWFPDSAFKTY  2026
             A+TVV GRARLGG+PVGV+AVETRTVE+ +PADPANLDSEAK +QQAGQVW+PDS++KT
Sbjct  2099  AKTVVTGRARLGGVPVGVIAVETRTVEVEMPADPANLDSEAKTLQQAGQVWYPDSSYKTA  2158

Query  2027  QAIKDFNREGLPLMVFANWRGFSGGMKDMYDQVLKFGAYIVDGLRECSQPVMVYIPPQAE  2086
             QAIKDF RE LPL+VFANWRGFSGGMKDMY+Q++KFGAYIVDGLRE  +PV++Y+PP AE
Sbjct  2159  QAIKDFGREELPLIVFANWRGFSGGMKDMYEQIVKFGAYIVDGLREYKKPVLIYLPPNAE  2218

Query  2087  LRGGSWVVIDPTINPRHMEMYADRESRGSVLEPEGTVEIKFRKKDLVKTMRRVDPVYIRL  2146
             LRGG+W V+D  INPR+ME YAD E+RG VLEPEG VEIK+++KDLVKT+ R+DP  I L
Sbjct  2219  LRGGAWAVLDSLINPRYMETYADPEARGGVLEPEGIVEIKYKEKDLVKTIHRLDPTTIAL  2278

Query  2147  AERLGTPELSPTERK--ELESKLKEREEFLIPIYHQVAVQFADLHDTPGRMQEKGVINDI  2204
              + L     S  + +  +++ K+K R   L+ +YH VAV FADLHDTP RM EK  I++I
Sbjct  2279  KKELDEANASGDKVRAAQVDEKIKARIAVLMHVYHTVAVHFADLHDTPERMLEKECISEI  2338

Query  2205  LDWKTSRTFFYWRLRRLLLEDLVKKKIHSANPELTDGQIQAMLRRWFVEVEGTVKAYVWD  2264
             + W+ SR + YWRLRRLLLED   KKI  A   L+ GQ + MLRRW VE +G  +AY+WD
Sbjct  2339  VPWRDSRRWLYWRLRRLLLEDAYIKKILRAQDNLSVGQAKQMLRRWLVEEKGATEAYLWD  2398
```

```
Query  2265  NNKDLVEWLEKQLTEEDGVRSVIEENIKYISRDYVLKQIRSLVQANPEVAMDSIVHMTQH  2324
              N+++V W E+Q+  E    S++  N+  + RD ++  I  +++  P+VA+D++V + Q
Sbjct  2399  KNEEMVSWYEEQINAE----SIVSRNVNSVRRDAIISTISKMLEDCPDVALDAVVGLCQG  2454

Query  2325  ISPTQRAEVVRILSTM  2340
             ++P  R  VVR L+ M
Sbjct  2455  LTPVNRGVVVRTLAQM  2470
```

Supplementary References

1. Abu-Elheiga, L. *et al.* The subcellular localization of acetyl-CoA carboxylase 2. *Proceedings of the National Academy of Sciences* **97**, 1444–1449 (2000).
2. Wang, Y. *et al.* Acetyl-CoA Carboxylases and Diseases. *Front. Oncol.* **12**, (2022).
3. Ivessa, A. S., Schneiter, R. & Kohlwein, S. D. Yeast acetyl-CoA carboxylase is associated with the cytoplasmic surface of the endoplasmic reticulum. *Eur J Cell Biol* **74**, 399–406 (1997).
4. Parvy, J.-P. *et al.* Drosophila melanogaster Acetyl-CoA-Carboxylase Sustains a Fatty Acid–Dependent Remote Signal to Waterproof the Respiratory System. *PLOS Genetics* **8**, e1002925 (2012).
5. Aso, Y. *et al.* Nitric oxide acts as a cotransmitter in a subset of dopaminergic neurons to diversify memory dynamics. *Elife* **8**, e49257 (2019).
6. Davie, K. *et al.* A Single-Cell Transcriptome Atlas of the Aging Drosophila Brain. *Cell* **174**, 982-998.e20 (2018).
