# Supplementary material for: Diverting glial glycolytic flux towards neurons is a memory-relevant role of Drosophila CRH-like signalling
Source: Nat Commun. 2024 Dec 2;15:10467. doi: 10.1038/s41467-024-54778-x (PMC11612226; doi:10.1038/s41467-024-54778-x)
Supplement: Supplementary file 2 — Reporting Summary [file 41467_2024_54778_MOESM2_ESM.pdf]

nature portfolio

Corresponding author(s): Pierre-Yves Plaçais, Thomas Preat

Last updated by author(s): Nov 13, 2024

Reporting Summary

Nature Portfolio wishes to improve the reproducibility of the work that we publish. This form provides structure for consistency and transparency in reporting. For further information on Nature Portfolio policies, see our Editorial Policies and the Editorial Policy Checklist.

Statistics

For all statistical analyses, confirm that the following items are present in the figure legend, table legend, main text, or Methods section.

n/a	Confirmed	
☐	☒	The exact sample size (*n*) for each experimental group/condition, given as a discrete number and unit of measurement
☐	☒	A statement on whether measurements were taken from distinct samples or whether the same sample was measured repeatedly
☐	☒	The statistical test(s) used AND whether they are one- or two-sided<br>*Only common tests should be described solely by name; describe more complex techniques in the Methods section.*
☒	☐	A description of all covariates tested
☐	☒	A description of any assumptions or corrections, such as tests of normality and adjustment for multiple comparisons
☐	☒	A full description of the statistical parameters including central tendency (e.g. means) or other basic estimates (e.g. regression coefficient) AND variation (e.g. standard deviation) or associated estimates of uncertainty (e.g. confidence intervals)
☐	☒	For null hypothesis testing, the test statistic (e.g. *F*, *t*, *r*) with confidence intervals, effect sizes, degrees of freedom and *P* value noted<br>*Give P values as exact values whenever suitable.*
☒	☐	For Bayesian analysis, information on the choice of priors and Markov chain Monte Carlo settings
☒	☐	For hierarchical and complex designs, identification of the appropriate level for tests and full reporting of outcomes
☐	☒	Estimates of effect sizes (e.g. Cohen's *d*, Pearson's *r*), indicating how they were calculated

Our web collection on statistics for biologists contains articles on many of the points above.

Software and code

Policy information about availability of computer code

Data collection	2-photon imaging: LAS-AF software v2.7.3 and LAS-X software v3.5.7 (Leica Microsystems); Immunostainings/Bodipy staining: NIS-Element v4.40 (Nikon)
Data analysis	Immunostainings: Fiji (Image J 1.52p); Lipid droplet analysis: Cell Profiler Analyst v3.1.9; Statistical testing: Prism 9.0 (Graphpad); 2-photon imaging analysis: LAS-X software v3.5.7 (Leica Microsystems)

For manuscripts utilizing custom algorithms or software that are central to the research but not yet described in published literature, software must be made available to editors and reviewers. We strongly encourage code deposition in a community repository (e.g. GitHub). See the Nature Portfolio guidelines for submitting code & software for further information.

Data

Policy information about availability of data

All manuscripts must include a data availability statement. This statement should provide the following information, where applicable:

- Accession codes, unique identifiers, or web links for publicly available datasets
- A description of any restrictions on data availability
- For clinical datasets or third party data, please ensure that the statement adheres to our policy

No datasets that require mandatory deposition into a public database were generated during the current study. Processed data from imaging experiments and raw data of behavioral assays, are provided in the Source data file. Unprocessed images, which represent a large volume, were not deposited in a public repository, as permanent storage by data centers raises increasing environmental and energy concerns. These data are available through e-mailing the corresponding authors,

and will be shared without restriction within a week. Source data are provided with this paper.

Research involving human participants, their data, or biological material

Policy information about studies with human participants or human data. See also policy information about sex, gender (identity/presentation), and sexual orientation and race, ethnicity and racism.

Reporting on sex and gender	N/A (research not involving human participants)
Reporting on race, ethnicity, or other socially relevant groupings	N/A (research not involving human participants)
Population characteristics	N/A (research not involving human participants)
Recruitment	N/A (research not involving human participants)
Ethics oversight	N/A (research not involving human participants)

Note that full information on the approval of the study protocol must also be provided in the manuscript.

Field-specific reporting

Please select the one below that is the best fit for your research. If you are not sure, read the appropriate sections before making your selection.

[x] Life sciences [ ] Behavioural & social sciences [ ] Ecological, evolutionary & environmental sciences

For a reference copy of the document with all sections, see nature.com/documents/nr-reporting-summary-flat.pdf

Life sciences study design

All studies must disclose on these points even when the disclosure is negative.

Sample size	No sample size calculation was performed. For both behavior and live imaging experiment, sample size was determined based on previous studies from our group : (e.g. refs. 38, 51,15). Sample size for lipid droplet measurement and qPCR was determined based on anotherprevious study from our group (ref. 17)
Data exclusions	In memory behavior experiments, the performance index was calculated as the number of flies attracted to the unconditioned odour minus the number of flies attracted to the conditioned odour, divided by the sum of the two numbers. To avoid giving disproportionate statistical importance to a small number of flies, measurements involving less than 6 flies in total were discarded.
Replication	All experiments were repeated in at least 2 independent experiments. In vivo imaging experiments were replicated in at least 3 independent experiments.
Randomization	Flies were assigned to experimental groups based on their genotypes.
Blinding	Investigators were not blinded, as for each experiment the same investigator performed crosses, data collection and analysis.

Reporting for specific materials, systems and methods

We require information from authors about some types of materials, experimental systems and methods used in many studies. Here, indicate whether each material, system or method listed is relevant to your study. If you are not sure if a list item applies to your research, read the appropriate section before selecting a response.

Materials & experimental systems

n/a	Involved in the study	
[ ]	[x]	Antibodies
[x]	[ ]	Eukaryotic cell lines
[x]	[ ]	Palaeontology and archaeology
[ ]	[x]	Animals and other organisms
[x]	[ ]	Clinical data
[x]	[ ]	Dual use research of concern
[x]	[ ]	Plants

Methods

n/a	Involved in the study	
[x]	[ ]	ChIP-seq
[x]	[ ]	Flow cytometry
[x]	[ ]	MRI-based neuroimaging

Antibodies

Antibodies used	anti-Dh44 antibody 1:1000 (source: Dr Jan Veenstra)<br>anti-elav 1:400 (DSHB 7E8A10)<br>anti-GFP 1:400 (Invitrogen, A11122)<br>The following secondary antibodies were used: anti-rabbit conjugated to Alexa Fluor 594 1:400 (Invitrogen, A11037), and anti-mouse conjugated to Alexa Fluor 488 1:400 (Invitrogen, A11029)
Validation	anti-Dh44: ref. 41 (Cabrero et al., 2002)<br>anti-GFP: https://antibodyregistry.org/search.php?q=AB_221569<br>anti-elav: https://www.antibodyregistry.org/AB_528218

Animals and other research organisms

Policy information about studies involving animals; ARRIVE guidelines recommended for reporting animal research, and Sex and Gender in Research

Laboratory animals	Experiments involved Drosophila melanogaster. The reference wild-type strain was Canton S. Experiments were performed on 1-4 day old adult flies. RNAi lines used in this study were KK108591 (RRID:Flybase_FBst0482273) and JF03208 (RRID:BDSC_28780) for Dh44-R1; JF03289 (RRID:BDSC_29610) for Dh44-R2; JF01822 (RRID:BDSC_25804) and KK110160 (RRID:Flybase_FBst0480283) for Dh44; GD3482 (RRID:Flybase_FBst0470958) for ACC; JF01188 (RRID:BDSC_31599) and JF01218 (RRID:BDSC_31277) for PKA-C1. Other fly lines were MB320C (RRID:BDSC_68253), NP2758-GAL4 (RRID:DGGR_104313), tub-GAL80ts (RRID:BDSC_7019), 13F02-LexA (RRID:BDSC_52460), 54H02-GAL4 (RRID:BDSC_45784), VT30559-GAL4 (RRID:Flybase_FBst0486483), Dh44-GAL4 (FBtp0129630), LexAop-Pyronic (FBtp0165803) and UAS-Pyronic (FBtp0141330).
Wild animals	No wild animal was used in the study
Reporting on sex	Both male and female flies were used for behaviour experiments. For in vivo imaging and immunostaining experiments, which involve dissection or surgery, female flies were assayed because of their larger size.
Field-collected samples	No field-collected samples were used in this study
Ethics oversight	No ethical approval was required for experiments done on Drosophila melanogaster.

Note that full information on the approval of the study protocol must also be provided in the manuscript.

Plants

Seed stocks	N/A
Novel plant genotypes	N/A
Authentication	N/A
